# A comparison of abundance estimates from extended batch-marking and Jolly–Seber-type experiments

**DOI:** 10.1002/ece3.899

**Published:** 2013-12-23

**Authors:** Laura L E Cowen, Panagiotis Besbeas, Byron J T Morgan, Carl J Schwarz

**Affiliations:** 1 Department of Mathematics and Statistics, University of VictoriaVictoria, British Columbia, Canada; 2 Department of Statistics, Athens University of Economics and BusinessAthens, Greece; 3 School of Mathematics, Statistics and Actuarial Science, University of KentCanterbury, Kent, U.K; 4 Department of Statistics and Actuarial Science, Simon Fraser UniversityBurnaby, British Columbia, Canada

**Keywords:** Abundance, batch mark, mark–recapture, open population

## Abstract

Little attention has been paid to the use of multi-sample batch-marking studies, as it is generally assumed that an individual's capture history is necessary for fully efficient estimates. However, recently, Huggins et al. (2010) present a pseudo-likelihood for a multi-sample batch-marking study where they used estimating equations to solve for survival and capture probabilities and then derived abundance estimates using a Horvitz–Thompson-type estimator. We have developed and maximized the likelihood for batch-marking studies. We use data simulated from a Jolly–Seber-type study and convert this to what would have been obtained from an extended batch-marking study. We compare our abundance estimates obtained from the Crosbie–Manly–Arnason–Schwarz (CMAS) model with those of the extended batch-marking model to determine the efficiency of collecting and analyzing batch-marking data. We found that estimates of abundance were similar for all three estimators: CMAS, Huggins, and our likelihood. Gains are made when using unique identifiers and employing the CMAS model in terms of precision; however, the likelihood typically had lower mean square error than the pseudo-likelihood method of Huggins et al. (2010). When faced with designing a batch-marking study, researchers can be confident in obtaining unbiased abundance estimators. Furthermore, they can design studies in order to reduce mean square error by manipulating capture probabilities and sample size.

## Introduction

Batch-marking experiments have largely been neglected by statistical ecologists, as they are deemed inferior and to be avoided (Pollock 1981; Pollock and Mann 1983). However, biologists still use batch-marking for various purposes, and for some studies, they may be the only option available (e.g., insects, juvenile fish).

There are other types of batch-marking studies that differ in design from the one we study here. For example, Measey et al. (2003) performed a three-sample batch-marking experiment on caecilians where individuals were given a batch mark on the first occasion and on the second occasion, a subsample was given a secondary mark. Both marked and unmarked captured individuals were recorded at each sample time. Because an individual's capture history can be deduced when a different batch mark is applied on each sampling occasion, a Jolly–Seber type model can be fitted to analyze these data (Jolly 1965; Seber 1965). However, the disadvantage of the design is that there is a physical limitation to how many marks can be applied to an individual and this would vary by both species and mark type.

Frequently, batch marks are used to study movement of individuals between locations. For example, Roberts and Angermeier (2007) studied the movements of three fish species in the South Fork of the Roanoke River, Virginia, using a two-sample study. Here, they constructed movement corridors with favorable pool characteristics between suitable habitats and compared movement rates in corridors with unfavorable characteristics. Captured fish were given a mark that was a randomly assigned color and body location. Recaptured individuals were counted, and movement rates were estimated.

Skalski et al. (2009) review several batch-marking designs and marking methods for very small fish. However, most of these result in complete capture history information and are for two or three sampling occasion designs.

Arguments against using batch marks are based on the lack of individual capture histories. For example, if a marked individual is captured at sample time three, it is not known whether this individual was one of the marked individuals captured at sample time two or not. In addition, batch-marking experiments do not allow for adequate testing of model assumptions (Williams et al. 2002; p. 312).

We motivate this work with the data found in Huggins et al. (2010). They describe a study of oriental weatherloach (*Misgurnus anguillicaudatu*), which is a freshwater fish native to Eurasia and Northern Africa. It was brought to Australia for use in aquaria but was accidentally released, and the aim of the study was to investigate activity patterns of the wild populations in the inland waters.

Huggins et al. (2010) provide a pseudo-likelihood method for analyzing an extended batch-marking study. They caution that the likelihood is intractable as the number of marked individuals alive at any sample time is unknown. They condition on released individuals to develop estimating equations and obtain capture and survival probability estimates. Then, they use a Horvitz–Thompson-type estimator to estimate population size at each time point after obtaining capture probability estimates. Standard errors are obtained by first using a sandwich estimator for the variance of the model parameters (Freedman 2012) and then using the delta method to obtain estimated standard errors for population size.

We develop the batch-marking likelihood conditional on release (rather than the pseudo-likelihood), followed by a Horvitz–Thompson-like estimator for abundance. Although theoretically the likelihood can be maximized, it involves nested summations, resulting in a large number of computations, but the calculations can be run in parallel when a multiprocessor computer, a cluster or a grid is available. For this article, we investigate the use of extended batch-marking data in comparison with the Crosbie–Manly–Arnason–Schwarz (CMAS) model (Schwarz and Arnason 1996) to study the loss in estimation precision when one does not have information on individual encounter histories for a seven sampling occasion simulation experiment under various parameter values.

## Materials and Methods

An extended batch-marking study is one where individuals captured at the first sample time are all given the same nonunique type of tag (e.g., blue in color). At subsequent sample times, individuals captured with tags are counted and unmarked individuals are given a different color batch mark resulting in an independent cohort. Table 1 provides an example of generated data from a four sampling occasion extended batch-marking experiment. New marks are not given to marked individuals, and thus individual capture histories cannot be obtained. For example, it is not known whether the nine blue-tagged individuals at sample time three are a subset of the 16 found at time two. Note the similarity to the m-array notation for Cormack–Jolly–Seber data (see Williams et al. 2002; p. 419).

**Table 1:** Example data for the extended bmarking design. The number of individuals marked with a particular tag color is found on the diagonal, while the number of recaptures is on the off diagonal.

		Occasion			
Release	Color	1	2	3	4
1	Blue	21	16	9	11
2	Green		22	15	12
3	Orange			17	4
4	Red				6

The assumptions we make are similar to other open population capture–recapture models namely:

- All individuals behave independently.
- All individuals have the same probability of capture at sample time *j*;*j* = 1, 2, …, *k*.
- All individuals have the same probability of survival between sample times *j* and *j* + 1; *j* = 1, 2, …, *k* − 1.
- Individuals do not lose their tags.

Below we detail notation used in the model development.

### Statistics or indices

*i* index for release occasion (or colour of tag).
*j* index for recapture occasion.
*k* the number of sampling occasions.
*r*_*ij*_ the number of individuals tagged and released at time *i* and recaptured at time *j*,*i* = 1, 2, …, *k* − 1; *j* = *i* + 1, …, *k*.
*R*_*i*_ the number of individuals released at time *i*;*i* = 1, 2, …, *k*.

### Latent variables

*M*_*ij*_ the number of marked individuals released at sample time *i*, alive and available for capture at sample time *j*;*j* = *i*,…, *k*. Note that *M*_*ii*_ = *R*_*i*_.
*d*_*ij*_ the number of deaths between sample times *j* and *j* + 1 from release group *i*;*d*_*ij*_* *= *M*_*ij*_ – *M*_*i,j+1*_; *i* = 1, …, *k*;*j* = *i*, …, *k* – 1.

### Parameters

*ϕ*_*ij*_ the probability of survival for individuals from release group *i* between times *j* and *j* + 1; *j* = 1, 2, …, *k* − 1.
*p*_*ij*_ the probability of capture for individuals from release group *i* at time *j*;*j* = 2, 3, …, *k*.

We develop the likelihood by first looking at the joint distribution of the recaptures *r*_*ij*_ and deaths *d*_*ij*_ given the releases *R*_*i*_. We then obtain the marginal distribution of the recaptures given releases by summing over all possible values of the deaths. The likelihood can be written as


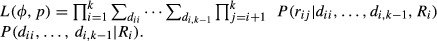
(1)

Conditional on release, we model the recaptures as independent given deaths 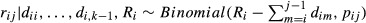 and the deaths as *d*_*ii*_, …,*d*_*ik*_|*R*_*i*_∼ *Multinomial*(*R*_*i*_, *π*_*ii*_, … ,*π*_*ik*_) where 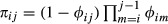. We note that 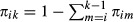 and 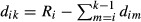 where *d*_*ik*_ would be the individuals that were released at time *i* and are still alive after the last sample time *k*. These *d*_*ik*_ are convenient for modeling purposes. Thus, the likelihood becomes


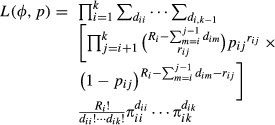
(2)

### Inference

The calculation of the likelihood involves nested summations for the latent *d*_*ij*_ variables which require high execution times if serially computed or cause the available RAM to be used up if fully vectorized. We developed parallel computer code to implement this model in MATLAB (MATLAB 2012) trading off CPU speed and memory that works for up to 11 sampling occasions. For experiments beyond 11 sampling occasions, we propose to use our likelihood up to the 11th sample time; and the pseudo-likelihood (Huggins et al. 2010) for occasions 12 through *k*. In the simulation studies, we first produce maximum likelihood estimates of the survival and capture probability parameters. Then, we derive a Horvitz–Thompson–type estimator for population size at each sample time (*N*_*j*_) using the capture probability estimates and the number of individuals captured at each sampling time, 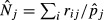. Standard errors for the 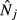 are estimated from the estimated variances of *r*_*ij*_ and 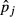 using the delta method (see Huggins et al. 2010 for details).

### Monte Carlo simulations

We simulated data from a *k* = 7 sample occasion Crosbie–Manly–Arnason–Schwarz model (Schwarz and Arnason 1996) with constant survival probabilities (*ϕ* = 0.2, 0.5, or 0.8), constant capture probabilities (*p* = 0.2, 0.5, or 0.8), and entry probabilities equal across time (1/*k*) with both a small superpopulation size (*N* = 200) and a larger superpopulation size (*N* = 1000). The superpopulation *N* is defined as the population that enters the population of interest at some point during the study. The computing time (on a dual quad core 2.53 GHz, 32 Gb RAM Linux server) was approximately 2 days to run 100 replications for *N* = 1000 but larger superpopulation size, more samples, or more replications would result in longer computing times. Parameter values were selected to obtain sparse-to-plentiful data by varying the probability of capture and survival.

For each set of parameter values, we simulated 100 CMAS datasets and collapsed these datasets into batch-marking data. We analyzed the individual capture history data using the CMAS model with constant parameters implemented in RMark (Laake 2013). The associated batch-marking data were analyzed using both the pseudo-likelihood (Huggins et al. 2010) and the likelihood with constant parameters (*ϕ*,*p*). For all methods of analyses, we estimated the survival and capture probabilities and obtained abundance estimates and estimated standard errors for each sampling time. The 100 dataset results are summarized using box plots for the estimated abundance, and estimated capture and survival probabilities. Root mean square errors (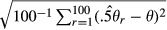) were calculated for the capture and survival probabilities. While standard errors were estimated, plots of these results are not included in the interest of space but are provided in the supplementary materials (see Supporting information).

## Results

**Figure 1:**
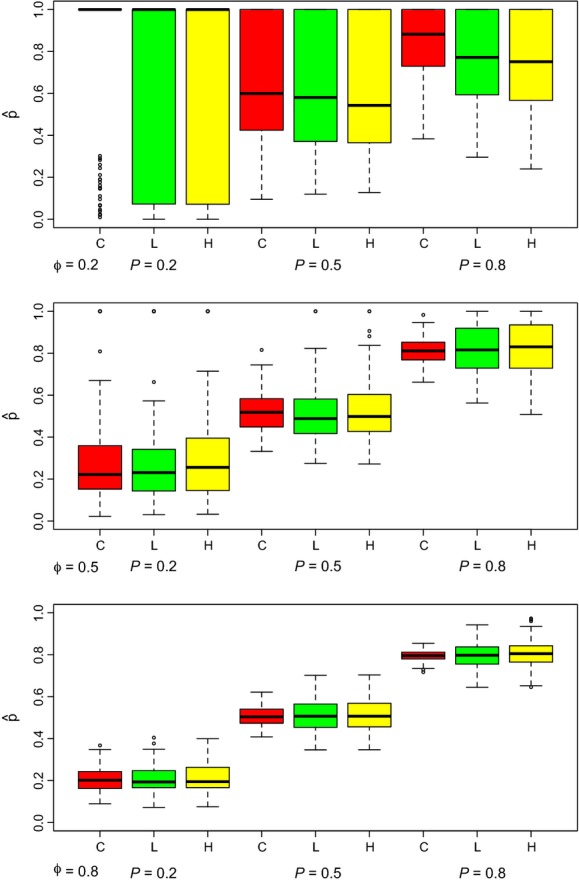
Boxplots of capture probability estimates (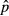) from 100 simulated datasets, for the Crosbie–Manly–Arnason–Schwarz (C: red), the likelihood (L: green), and the pseudo-likelihood (H: yellow; Huggins et al. 2010) methods when parameter values are *N* = 200, and *p* = 0.2, 0.5, 0.8 for *ϕ* = 0.2 (top), *ϕ* = 0.5 (middle), and *ϕ* = 0.8 (bottom).

**Figure 2:**
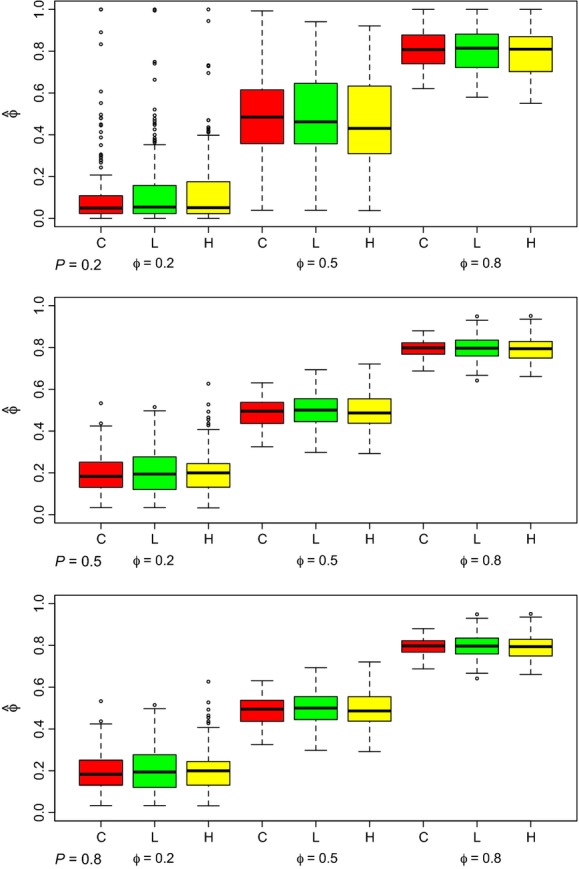
Boxplots of survival probability estimates (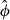) from 100 simulated datasets, for the Crosbie–Manly–Arnason–Schwarz (C: red), the likelihood (L: green), and the pseudo-likelihood (H: yellow; Huggins et al. 2010) methods when parameter values are *N* = 200, and *ϕ* = 0.2, 0.5, 0.8 for *p* = 0.2 (top), *p* = 0.5 (middle), and *p* = 0.8 (bottom).

Figures 1 and 2 provide results for the 9 simulation studies under varying parameter values for estimates of *p* and *ϕ*, respectively, for *N* = 200. For sparse data (*p* = 0.2 or *p* = 0.5, *ϕ* = 0.2), many of the simulations produced boundary estimates for *p* and occasionally *ϕ* (see Fig. 1), and the calculation of the standard errors then failed due to the Hessian being singular. These simulation failures are similar to what happens in the Cormack–Jolly–Seber model when analytical estimates of *ϕ* exceed 1 with sparse data and actual parameter values are close to 0 and 1. In these cases, the maximization function in MATLAB constrained estimates to be admissible, i.e. between 0 and 1 (inclusive). When the estimation of *p* was on a boundary, *N*_*j*_ was estimated at infinity (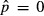) or ∑_*i*_*r*_*ij*_ (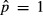). Table2 provides the number of simulations out of 100 that produced boundary estimates for *p* or *ϕ* for all three methods. Similar figures for *N* = 1000 are provided in the Supporting information. Results for the estimation of standard errors are based on those simulations that did not fail (see Supporting information).

**Table 2:** The number of simulations out of 100 that produced boundary estimates (0 or 1) for either parameter *p* or *ϕ* for the Crosbie–Manly–Arnason–Schwarz (CMAS), the likelihood (L), and the pseudo-likelihood (H; Huggins et al. 2010) methods under the 18 simulation scenarios.

			*ϕ*		
*N*	Method	*p*	0.2	0.5	0.8
200	CMAS	0.2	95	9	2
		0.5	32	0	0
		0.8	37	0	0
	L	0.2	75	9	6
		0.5	32	2	0
		0.8	36	18	0
	H	0.2	76	10	6
		0.5	26	2	0
		0.8	31	16	0
1000	CMAS	0.2	32	0	0
		0.5	1	0	1
		0.8	1	0	0
	L	0.2	27	0	0
		0.5	3	0	0
		0.8	9	2	0
	H	0.2	24	0	0
		0.5	8	0	0
		0.8	14	1	0

The root mean square error (RMSE) for estimates of *p* and *ϕ* are given in Tables3 and 4, respectively. As expected, we find that within a method, RMSE decreases as *p* and *ϕ* increase. Similarly, RMSE decreases with increased *N*. We also confirm that the CMAS method typically has lower RMSE than either of the batch marking methods and that the likelihood method typically has lower RMSE than the pseudo-likelihood method. Exceptions to this occur with sparse data when the estimates are not reliable.

**Table 3:** Root mean square error for estimates of *p* for the Crosbie–Manly–Arnason–Schwarz (CMAS), the likelihood (L), and the pseudo-likelihood (H; Huggins et al. 2010) methods under the 18 simulation scenarios.

			*ϕ*					
*N*	Method	*p*	0.2	0.5	0.8
200	CMAS	0.2	0.704	0.282	0.062
		0.5	0.319	0.100	0.046
		0.8	0.160	0.065	0.028
	L	0.2	0.616	0.271	0.065
		0.5	0.330	0.136	0.077
		0.8	0.215	0.123	0.058
	H	0.2	0.616	0.296	0.073
		0.5	0.309	0.151	0.079
		0.8	0.233	0.125	0.068
1000	CMAS	0.2	0.470	0.066	0.026
		0.5	0.149	0.036	0.019
		0.8	0.086	0.027	0.011
	L	0.2	0.448	0.083	0.035
		0.5	0.185	0.050	0.031
		0.8	0.137	0.057	0.031
	H	0.2	0.428	0.087	0.035
		0.5	0.212	0.053	0.032
		0.8	0.150	0.061	0.034

**Table 4:** Root mean square error for estimates of *ϕ* for the Crosbie–Manly–Arnason–Schwarz (CMAS), the likelihood (L), and the pseudo-likelihood (H; Huggins et al. 2010) methods under the 18 simulation scenarios.

			*ϕ*		
*N*	Method	*p*	0.2	0.5	0.8
200	CMAS	0.2	0.224	0.196	0.095
		0.5	0.087	0.068	0.040
		0.8	0.041	0.036	0.027
	L	0.2	0.221	0.197	0.106
		0.5	0.101	0.083	0.058
		0.8	0.064	0.053	0.035
	H	0.2	0.215	0.206	0.116
		0.5	0.112	0.090	0.060
		0.8	0.075	0.060	0.039
1000	CMAS	0.2	0.121	0.072	0.046
		0.5	0.038	0.027	0.014
		0.8	0.021	0.016	0.009
	L	0.2	0.116	0.093	0.067
		0.5	0.049	0.035	0.021
		0.8	0.035	0.025	0.016
	H	0.2	0.116	0.096	0.067
		0.5	0.053	0.037	0.023
		0.8	0.038	0.027	0.017

**Figure 3:**
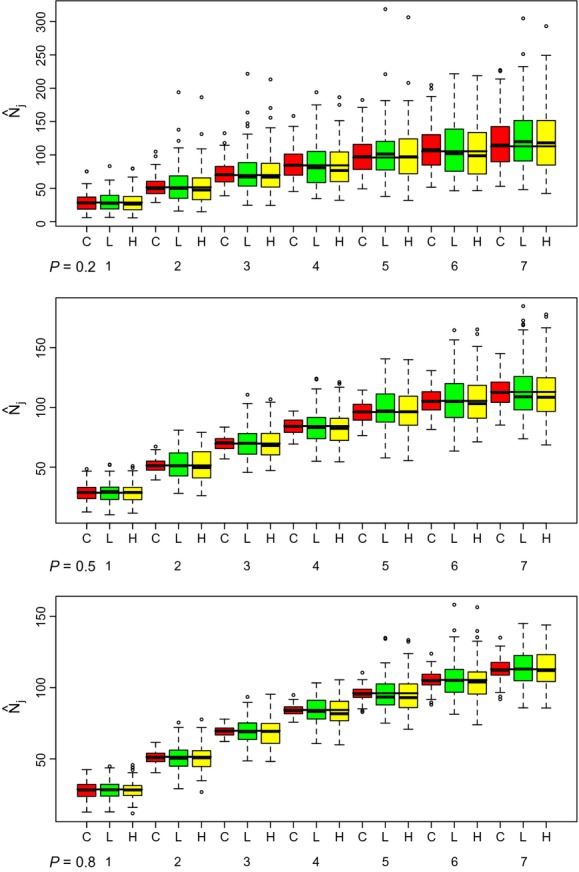
Boxplots of abundance estimates (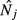) for each sample time (*k* = 7) from 100 simulated datasets, for the Crosbie–Manly–Arnason–Schwarz (C: red), the likelihood (L: green), and the pseudo-likelihood (H: yellow; Huggins et al. 2010) methods when parameter values are *N* = 200, and *ϕ* = 0.8 for *p* = 0.2 (top), *p* = 0.5 (middle), and *p* = 0.8 (bottom). The long black horizontal lines show the expected population size at time *j*.

Under sparse data conditions (e.g., *N* = 200, *ϕ* = 0.8, *p* = 0.2), the average population size estimates are similar between the three methods; however, variability in estimates is higher for the likelihood and pseudo-likelihood methods as expected (Fig. 3; box plots for other sets of parameters are provided in Supporting information). For example, average population size estimates for time three were 71, 74, and 72 individuals for the CMAS, likelihood, and pseudo-likelihood, respectively, and the corresponding average standard error estimates were 18, 32, and 32 individuals. For higher quality data (e.g., *N* = 1000, *p* = 0.5, *ϕ* = 0.8), we found similar results. The CMAS model produces more precise estimates followed by the likelihood (see Supporting information for box plots of estimated standard errors). For example, the average population size estimate for sample time three was 348, 349, and 349 individuals for the CMAS, likelihood, and pseudo-likelihood method, respectively, with corresponding average estimated standard errors of 12, 29, and 29 individuals.

## Discussion

For an extended batch-marking study, using the likelihood provides more accurate estimates and lower standard errors than using the pseudo-likelihood method of Huggins et al. (2010). However, the computing power necessary to calculate the likelihood by summing over all possible values of deaths is prohibitive when sampling times go beyond *k* = 11 or if *R*_*i*_ is large. In these cases, the pseudo-likelihood method is computationally faster and provides unbiased estimates with similar precision to the likelihood approach. Ultimately, if full-capture histories are possible and available, then naturally the CMAS model outperforms both batch-marking models. With plentiful data (large numbers of recaptures), our model can have a relative efficiency of between about 30–40% compared with the CMAS model; thus, using the CMAS model has obvious gains in precision.

In many of the plots for 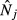, the average population size increases over time. This is due to the models allowing for births/immigration into the population from the superpopulation (*N*). In these simulations, entry probabilities were equal across time and summed to one. Thus, with high survival rates, population size would naturally increase with time.

Practitioners who are confined to using batch marks should design their studies to have large sample sizes and high capture rates so as to minimize mean square error.

For future work, we will complete the model development by incorporating both tagged and untagged individuals at each sample time. We will also deal with issues such as goodness of fit, model selection, and parameter redundancy. With the many latent variables in the complete data, this model lends itself well to Bayesian methods where a state-space formulation is under development.

With permanent batch marks, tag loss would not be an issue. However, if injectable color tags are used for example, tag loss may bias parameter estimates. If it were possible to double tag individuals, an extended batch-marking model incorporating tag retention rates could be developed using methods similar to Cowen and Schwarz (2006). However, for those study species where double tagging is not possible (e.g., insects), separate experiments to estimate tag retention would have to be carried out and this auxiliary information could be used to adjust parameter estimates using methods similar to Arnason and Mills (1981).

## Supporting Information

**Figure S1.** Boxplots of capture probability estimates () of 100 simulated datasets, for the Crosbie–Manley–Arnason–Schwarz (C: red), the likelihood (L: green), and the pseudo-likelihood (H: yellow; Huggins et al. 2010) when parameters values are N = 1000, and *p* = 0.2,0.5,0.8 for ϕ = 0.2 (top), and ϕ = 0.5 (middle), ϕ = 0.8 (bottom). **Figure S2.** Boxplots of survival probability estimates  of 100 simulated datasets, for the Crosbie–Manley–Arnason–Schwarz (C: red), the likelihood (L: green), and the pseudo-likelihood (H: yellow; Huggins et al. 2010) when parameters values are N = 1000, and ϕ = 0.2, 0.5, 0.8 for *p* = 0.2 (top), and *p* = 0.5 (middle), *p* = 0.8 (bottom). **Figure S3.** Boxplots of abundance estimates  for each sample time (k = 7) of 100 simulated datasets, for the Crosbie–Manley–Arnason–Schwarz (C: red), the likelihood (L: green), and the pseudo-likelihood (H: yellow; Huggins et al. 2010) when parameters values are N = 200, and ϕ = 0.2 for *p* = 0.2 (top), and *p* = 0.5 (middle), *p* = 0.8 (bottom). The long black horizontal lines show the expected population size at time *j*. **Figure S4.** Boxplots of abundance estimates  for each sample time (k = 7) of 100 simulated datasets, for the Crosbie–Manley–Arnason–Schwarz (C: red), the likelihood (L: green), and the pseudo-likelihood (H: yellow; Huggins et al. 2010) when parameters values are N = 200, and ϕ = 0.5 for *p* = 0.2 (top), and *p* = 0.5 (middle), *p* = 0.8 (bottom). The long black horizontal lines show the expected population size at time *j*. **Figure S5.** Boxplots of abundance estimates  for each sample time (k = 7) of 100 simulated datasets, for the Crosbie–Manley–Arnason–Schwarz (C: red), the likelihood (L: green), and the pseudo-likelihood (H: yellow; Huggins et al. 2010) when parameters values are N = 1000, and ϕ = 0.2 for *p* = 0.2 (top), and *p* = 0.5 (middle), *p* = 0.8 (bottom). The long black horizontal lines show the expected population size at time *j*. **Figure S6.** Boxplots of abundance estimates  for each sample time (k = 7) of 100 simulated datasets, for the Crosbie–Manley–Arnason–Schwarz (C: red), the likelihood (L: green), and the pseudo-likelihood (H: yellow; Huggins et al. 2010) when parameters values are N = 1000, and ϕ = 0.5 for *p* = 0.2 (top), and *p* = 0.5 (middle), *p* = 0.8(bottom). The long black horizontal lines show the expected population size at time *j*. **Figure S7.** Boxplots of abundance estimates  for each sample time (k = 7) of 100 simulated datasets, for the Crosbie–Manley–Arnason–Schwarz (C: red), the likelihood (L: green), and the pseudo-likelihood (H: yellow; Huggins et al. 2010) when parameters values are N = 1000, and ϕ = 0.8 for *p* = 0.2 (top), and *p* = 0.5 (middle), *p* = 0.8 (bottom). The long black horizontal lines show the expected population size at time *j*. **Figure S8.** Boxplots of estimated standard errors for the abundance estimates () for each sample time (k = 7) of 100 simulated datasets, for the Crosbie–Manley–Arnason–Schwarz (C: red), the likelihood (L: green), and the pseudo-likelihood (H: yellow; Huggins et al. 2010) when parameters values are N = 200, and ϕ = 0.2 for *p* = 0.2 (top), and *p* = 0.5 (middle), *p* = 0.8 (bottom). Estimates from simulations that produced a singular Hessian were removed. **Figure S9.** Boxplots of estimated standard errors for the abundance estimates () for each sample time (k = 7) of 100 simulated datasets, for the Crosbie–Manley–Arnason–Schwarz (C: red), the likelihood (L: green), and the pseudo-likelihood (H: yellow; Huggins et al. 2010) when parameters values are N = 200, and ϕ = 0.5 for *p* = 0.2 (top), and *p* = 0.5 (middle), *p* = 0.8 (bottom). Estimates from simulations that produced a singular Hessian were removed. **Figure S10.** Boxplots of estimated standard errors for the abundance estimates () for each sample time (k = 7) of 100 simulated datasets, for the Crosbie–Manley–Arnason–Schwarz (C: red), the likelihood (L: green), and the pseudo-likelihood (H: yellow; Huggins et al. 2010) when parameters values are N = 200, and ϕ = 0.8 for *p* = 0.2 (top), and *p* = 0.5 (middle), *p* = 0.8 (bottom). Estimates from simulations that produced a singular Hessian were removed. **Figure S11.** Boxplots of estimated standard errors for the abundance estimates () for each sample time (k = 7) of 100 simulated datasets, for the Crosbie–Manley–Arnason–Schwarz (C: red), the likelihood (L: green), and the pseudo-likelihood (H: yellow; Huggins et al. 2010) when parameters values are N = 1000, and ϕ = 0.2 for *p* = 0.2 (top), and *p* = 0.5 (middle), *p* = 0.8 (bottom). Estimates from simulations that produced a singular Hessian were removed. **Figure S12.** Boxplots of estimated standard errors for the abundance estimates () for each sample time (k = 7) of 100 simulated datasets, for the Crosbie–Manley–Arnason–Schwarz (C: red), the likelihood (L: green), and the pseudo-likelihood (H: yellow; Huggins et al. 2010) when parameters values are N = 1000, and ϕ = 0.5 for *p* = 0.2 (top), and *p* = 0.5 (middle), *p* = 0.8 (bottom). Estimates from simulations that produced a singular Hessian were removed. **Figure S13.** Boxplots of estimated standard errors for the abundance estimates () for each sample time (k = 7) of 100 simulated datasets, for the Crosbie–Manley–Arnason–Schwarz (C: red), the likelihood (L: green), and the pseudo-likelihood (H: yellow; Huggins et al. 2010) when parameters values are N = 1000, and ϕ = 0.8 for *p* = 0.2 (top), and *p* = 0.5 (middle), *p* = 0.8 (bottom). Estimates from simulations that produced a singular Hessian were removed.
