# Supplementary material for: A comparison of abundance estimates from extended batch-marking and Jolly–Seber-type experiments
Source: Ecol Evol. 2013 Dec 23;4(2):210–8. doi: 10.1002/ece3.899 (PMC3925384; doi:10.1002/ece3.899)
Supplement: Supplementary file 1 — Figure S1. Boxplots of capture probability estimates () of 100 simulated datasets, for the Crosbie–Manley–Arnason–Schwarz (C: red), the likelihood (L: green), and the pseudo-likelihood (H: yellow; Huggins et al. 2010) when parameters values are N = 1000, and p = 0.2,0.5,0.8 for ϕ = 0.2 (top), and ϕ = 0.5 (middle), ϕ = 0.8 (bottom). Figure S2. Boxplots of survival probability estimates of 100 simulated datasets, for the Crosbie–Manley–Arnason–Schwarz (C: red), the likelihood (L: green), and the pseudo-likelihood (H: yellow; Huggins et al. 2010) when parameters values are N = 1000, and ϕ = 0.2, 0.5, 0.8 for p = 0.2 (top), and p = 0.5 (middle), p = 0.8 (bottom). Figure S3. Boxplots of abundance estimates for each sample time (k = 7) of 100 simulated datasets, for the Crosbie–Manley–Arnason–Schwarz (C: red), the likelihood (L: green), and the pseudo-likelihood (H: yellow; Huggins et al. 2010) when parameters values are N = 200, and ϕ = 0.2 for p = 0.2 (top), and p = 0.5 (middle), p = 0.8 (bottom). The long black horizontal lines show the expected population size at time j. Figure S4. Boxplots of abundance estimates for each sample time (k = 7) of 100 simulated datasets, for the Crosbie–Manley–Arnason–Schwarz (C: red), the likelihood (L: green), and the pseudo-likelihood (H: yellow; Huggins et al. 2010) when parameters values are N = 200, and ϕ = 0.5 for p = 0.2 (top), and p = 0.5 (middle), p = 0.8 (bottom). The long black horizontal lines show the expected population size at time j. Figure S5. Boxplots of abundance estimates for each sample time (k = 7) of 100 simulated datasets, for the Crosbie–Manley–Arnason–Schwarz (C: red), the likelihood (L: green), and the pseudo-likelihood (H: yellow; Huggins et al. 2010) when parameters values are N = 1000, and ϕ = 0.2 for p = 0.2 (top), and p = 0.5 (middle), p = 0.8 (bottom). The long black horizontal lines show the expected population size at time j. Figure S6. Boxplots of abundance estimates for each sample time (k = 7) of 10 [file ece30004-0210-sd1.pdf]

A comparison of abundance estimates from extended batch-marking and Jolly-Seber type experiments: Supplementary material.

Cowen, L. L. E, Besbeas, P., Morgan, B. J. T., and Schwarz, C. J.

Here we provide the plots that resulted from our $k = 7$ sample time simulation study with parameter values of $p = 0.2, 0.5, 0.8$; $\phi = 0.2, 0.5, 0.8$; and $N = 200, 1000$ that were not presented in the main manuscript.

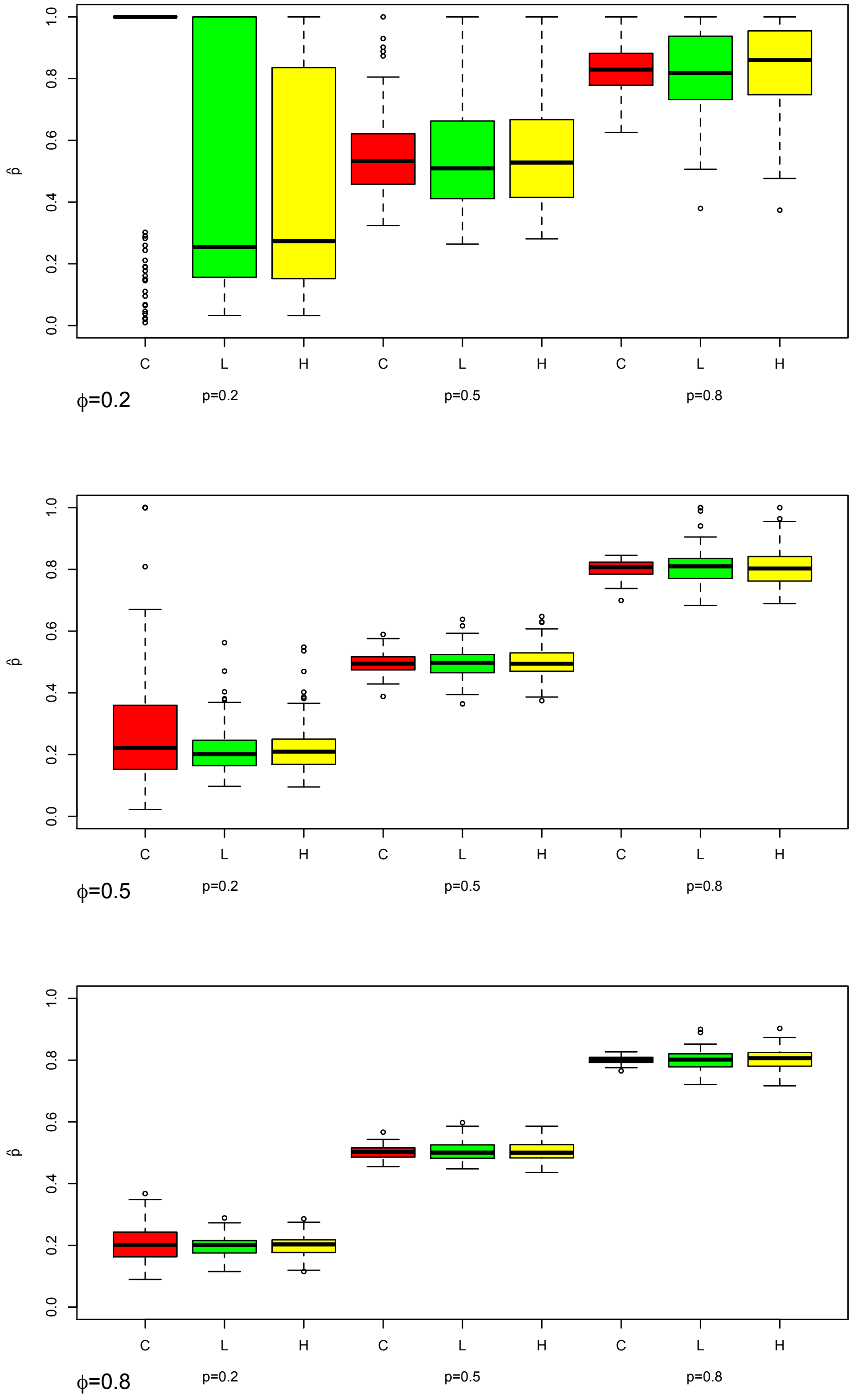

Figure 1: Capture probability estimates ($\hat{p}$) of 100 simulated datasets, for the Crosbie-Manley-Arnason-Schwarz (C: red), the likelihood (L: green), and the pseudo-likelihood (H: yellow; Huggins *et al.* 2010) when parameters values are $N = 1000$, and $p = 0.2, 0.5, 0.8$ for $\phi = 0.2$ (top), $\phi = 0.5$ (middle), and $\phi = 0.8$ (bottom).

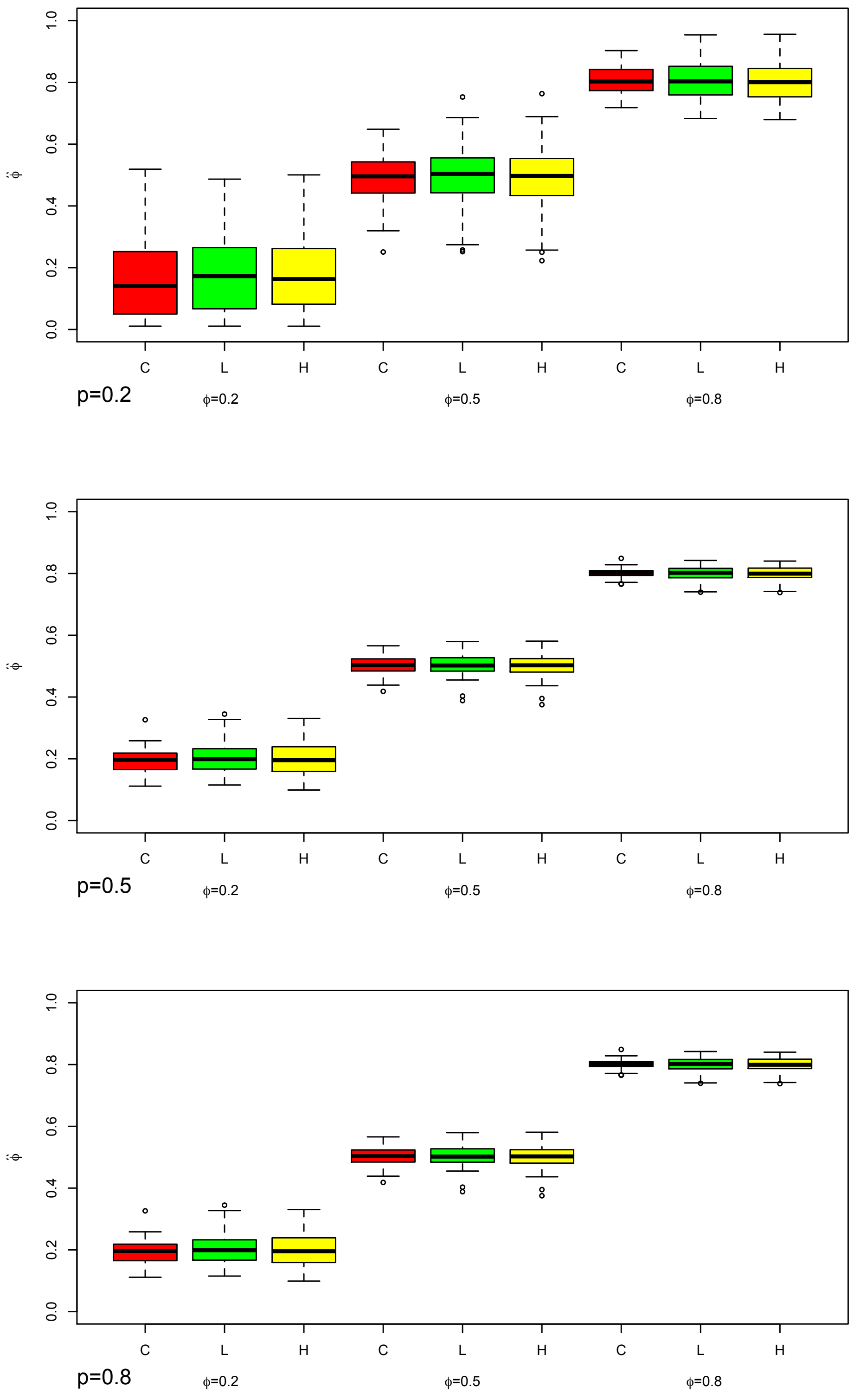

Figure 2: Survival probability estimates ($\hat{\phi}$) of 100 simulated datasets, for the Crosbie-Manley-Arnason-Schwarz (C: red), the likelihood (L: green), and the pseudo-likelihood (H: yellow; Huggins *et al.* 2010) when parameters values are $N = 1000$, and $\phi = 0.2, 0.5, 0.8$ for $p = 0.2$ (top), $p = 0.5$ (middle), and $p = 0.8$ (bottom).

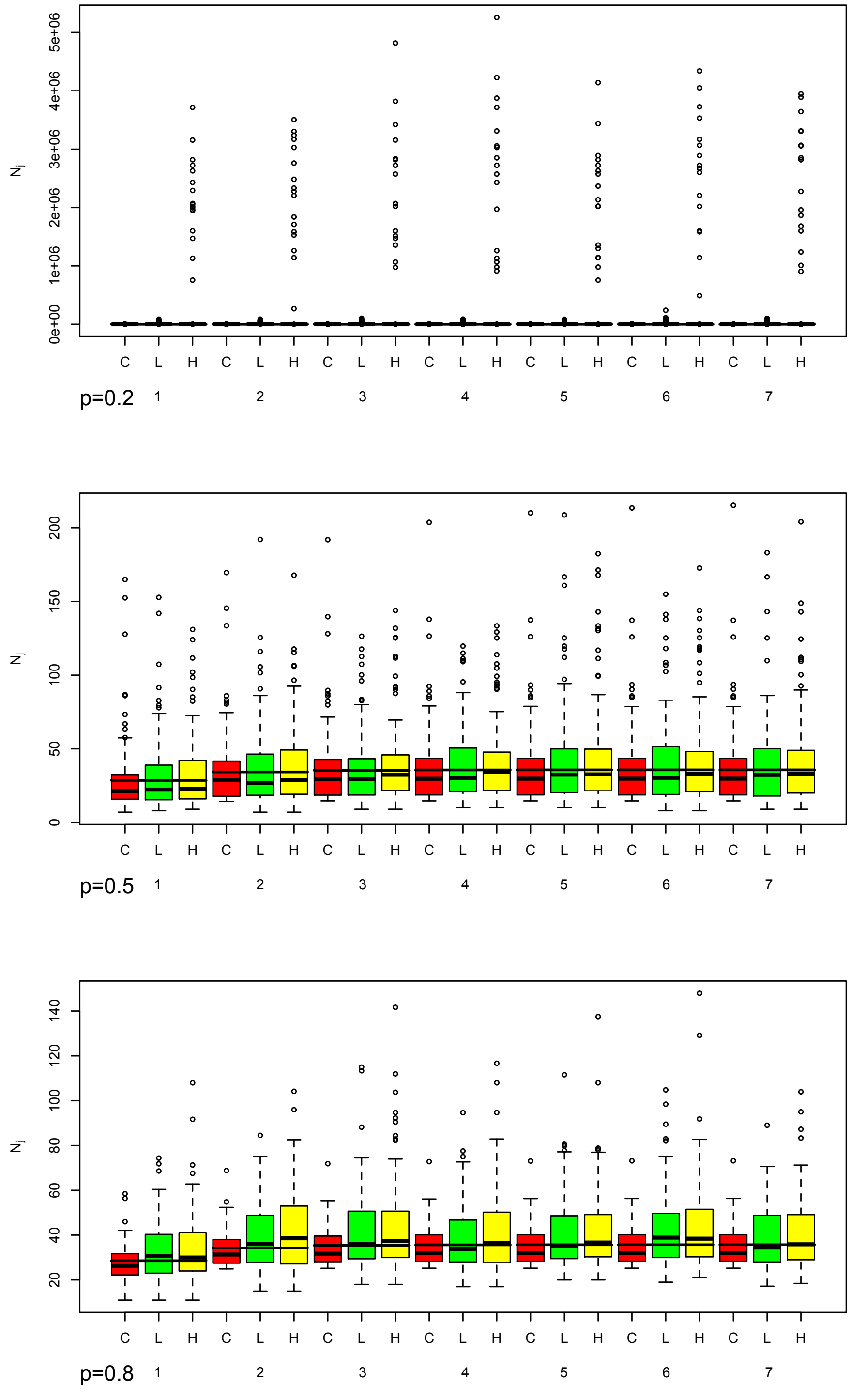

Figure 3: Abundance estimates ($\hat{N}_j$) for each sample time ($k = 7$) of 100 simulated datasets, for the Crosbie-Manley-Arnason-Schwarz (C: red), the likelihood (L: green), and the pseudo-likelihood (H: yellow; Huggins *et al.* 2010) when parameters values are $N = 200$, and $\phi = 0.2$ for $p = 0.2$ (top), $p = 0.5$ (middle), and $p = 0.8$ (bottom). The long black horizontal lines show the expected population size at time j.

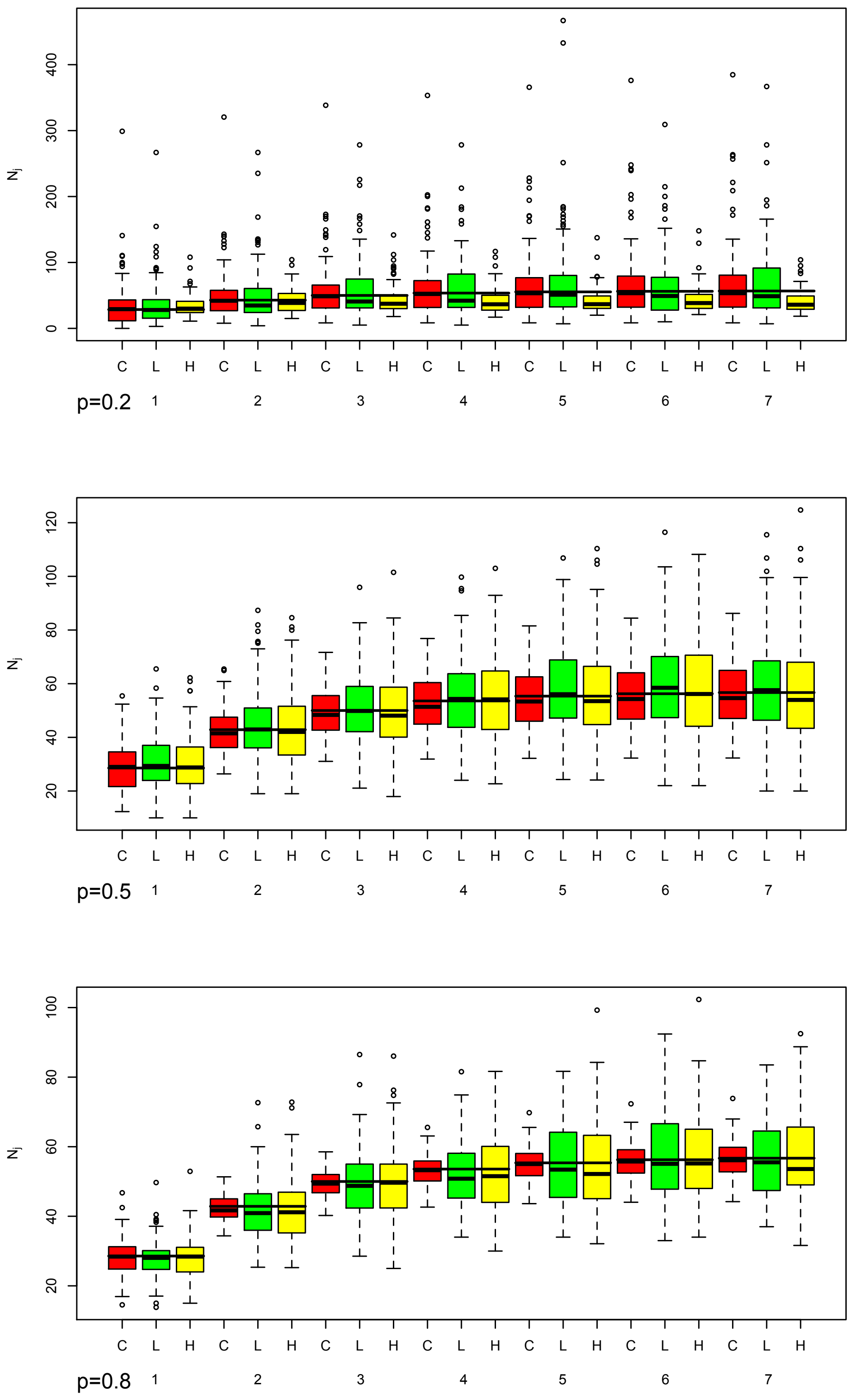

Figure 4: Abundance estimates ($\hat{N}_j$) for each sample time ($k = 7$) of 100 simulated datasets, for the Crosbie-Manley-Arnason-Schwarz (C: red), the likelihood (L: green), and the pseudo-likelihood (H: yellow; Huggins *et al.* 2010) when parameters values are $N = 200$, and $\phi = 0.5$ for $p = 0.2$ (top), $p = 0.5$ (middle), and $p = 0.8$ (bottom). The long black horizontal lines show the expected population size at time j.

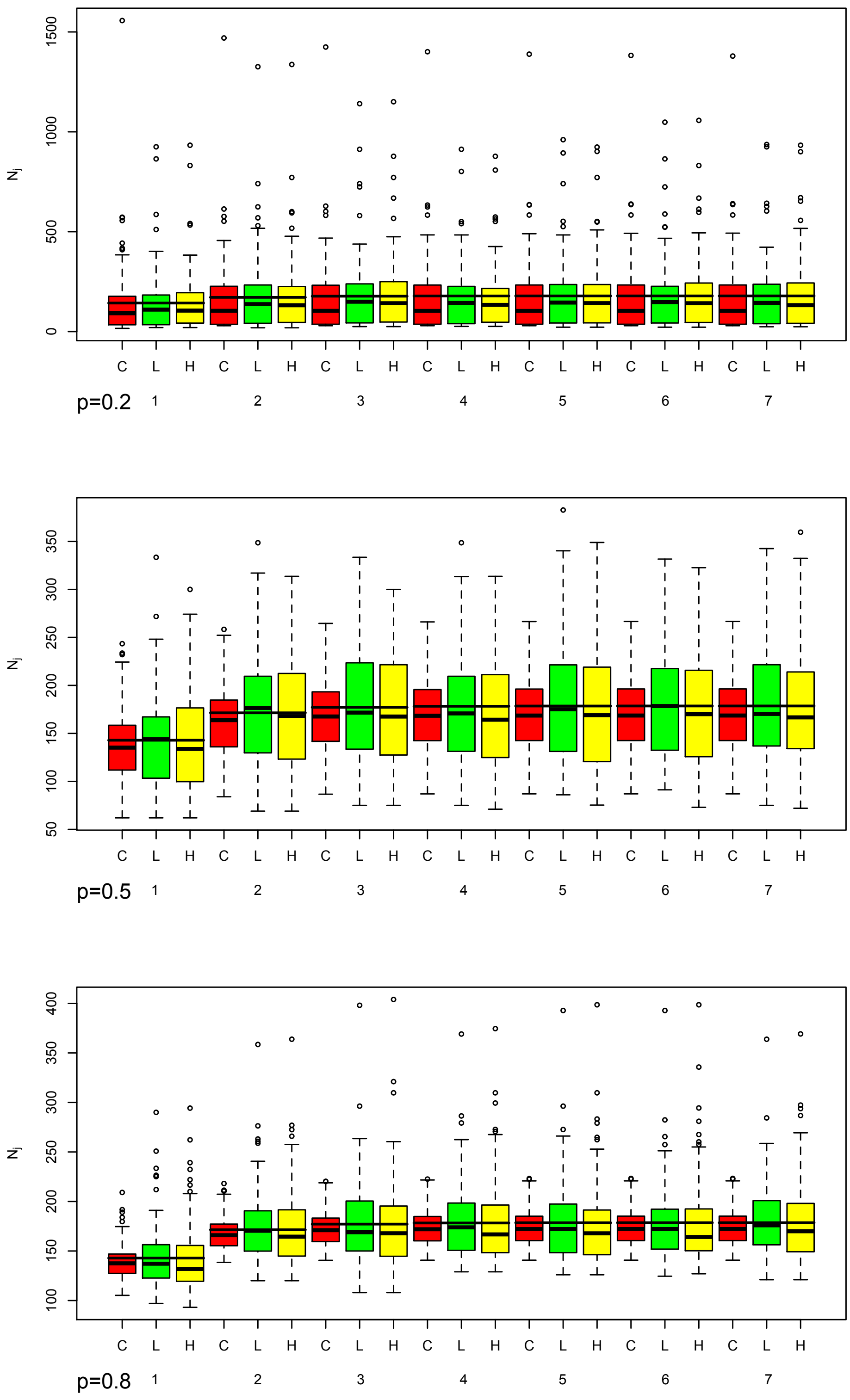

Figure 5: Abundance estimates ($\hat{N}_j$) for each sample time ($k = 7$) of 100 simulated datasets, for the Crosbie-Manley-Arnason-Schwarz (C: red), the likelihood (L: green), and the pseudo-likelihood (H: yellow; Huggins *et al.* 2010) when parameters values are $N = 1000$, and $\phi = 0.2$ for $p = 0.2$ (top), $p = 0.5$ (middle), and $p = 0.8$ (bottom). The long black horizontal lines show the expected population size at time j.

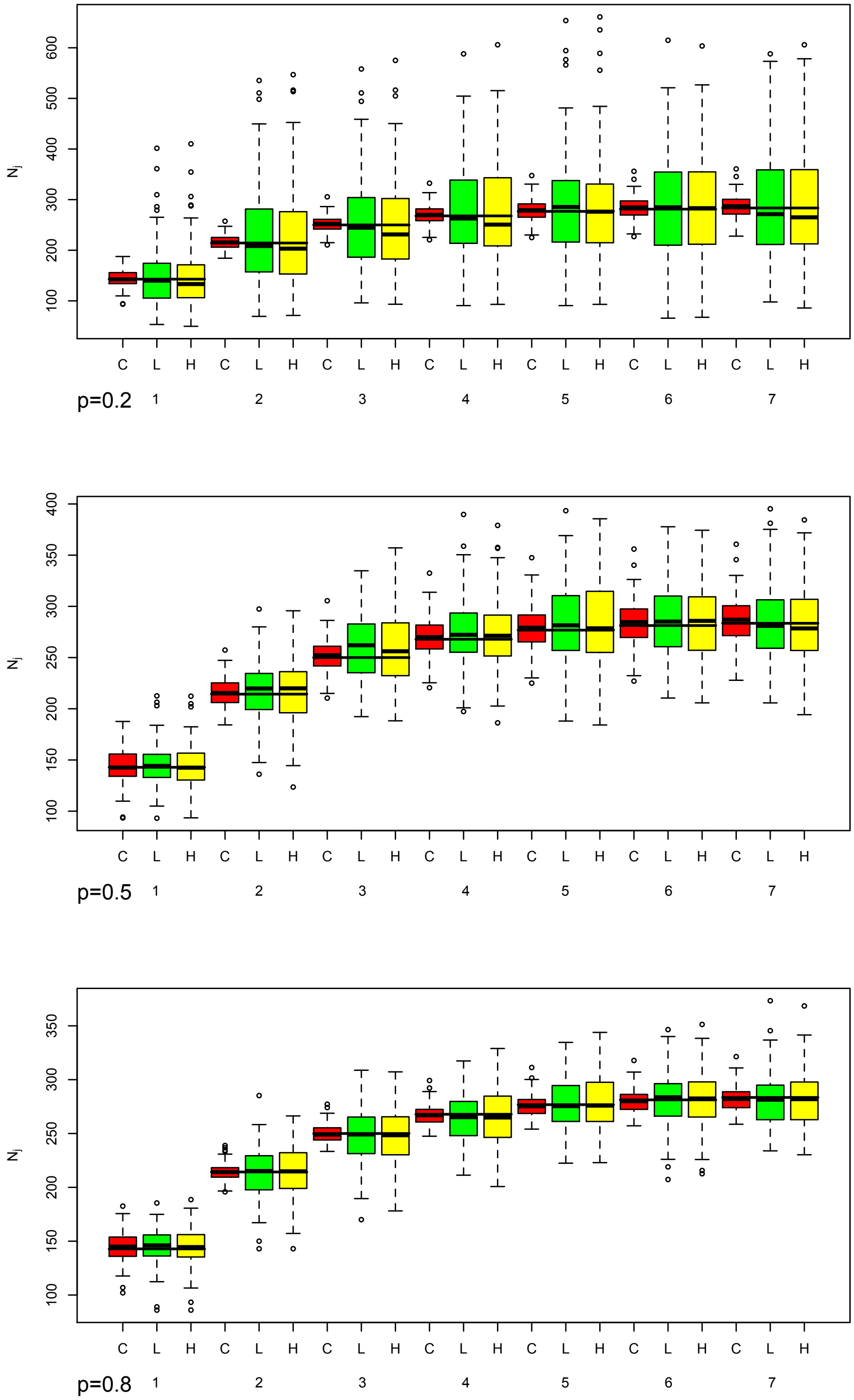

Figure 6: Abundance estimates $(\hat{N}_j)$ for each sample time $(k = 7)$ of 100 simulated datasets, for the Crosbie-Manley-Arnason-Schwarz (C: red), the likelihood (L: green), and the pseudo-likelihood (H: yellow; Huggins *et al.* 2010) when parameters values are $N = 1000$, and $\phi = 0.5$ for $p = 0.2$ (top), $p = 0.5$ (middle), and $p = 0.8$ (bottom). The long black horizontal lines show the expected population size at time j.

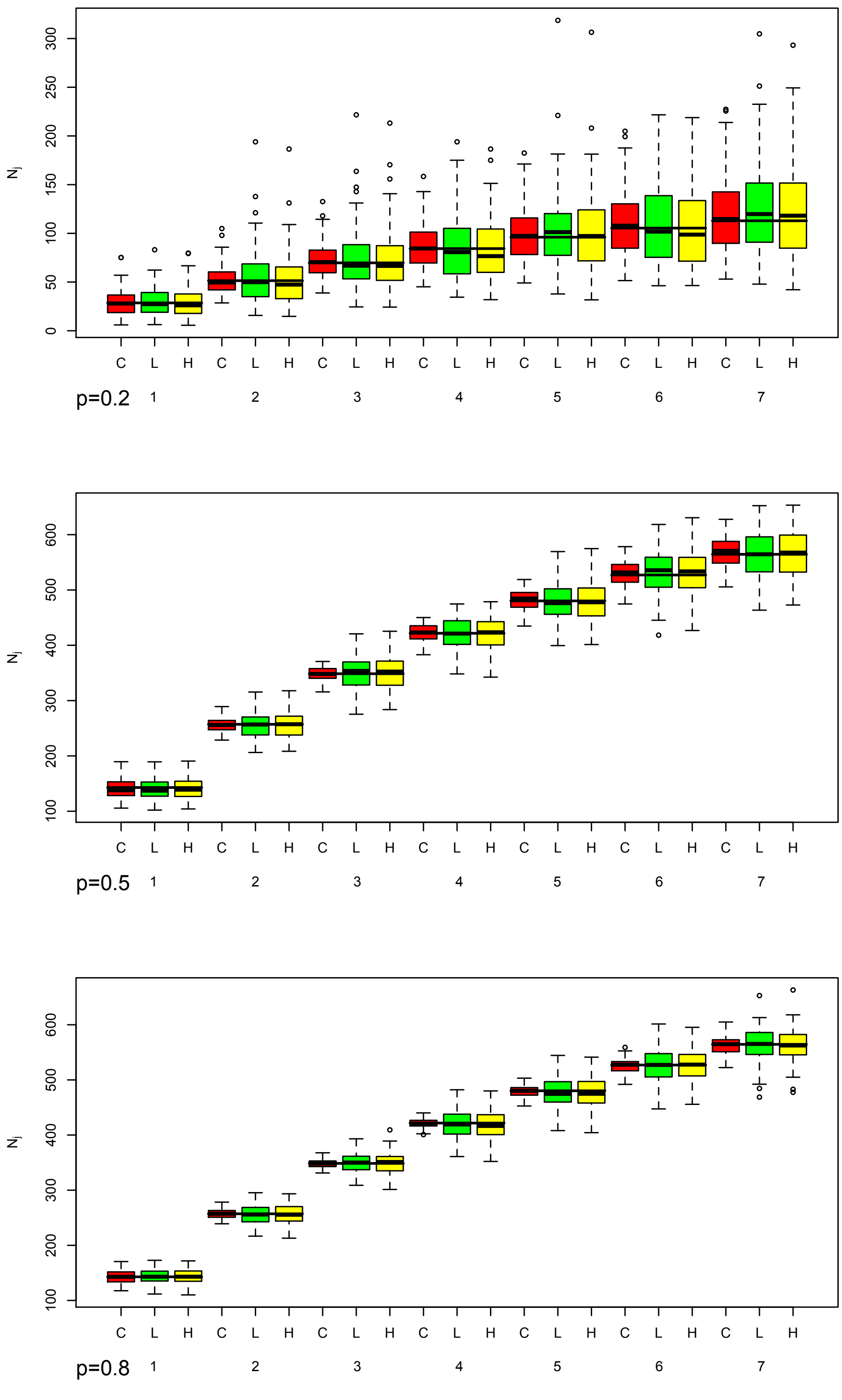

Figure 7: Abundance estimates ($\hat{N}_j$) for each sample time ($k = 7$) of 100 simulated datasets, for the Crosbie-Manley-Arnason-Schwarz (C: red), the likelihood (L: green), and the pseudo-likelihood (H: yellow; Huggins *et al.* 2010) when parameters values are $N = 1000$, and $\phi = 0.8$ for $p = 0.2$ (top), $p = 0.5$ (middle), and $p = 0.8$ (bottom). The long black horizontal lines show the expected population size at time j.

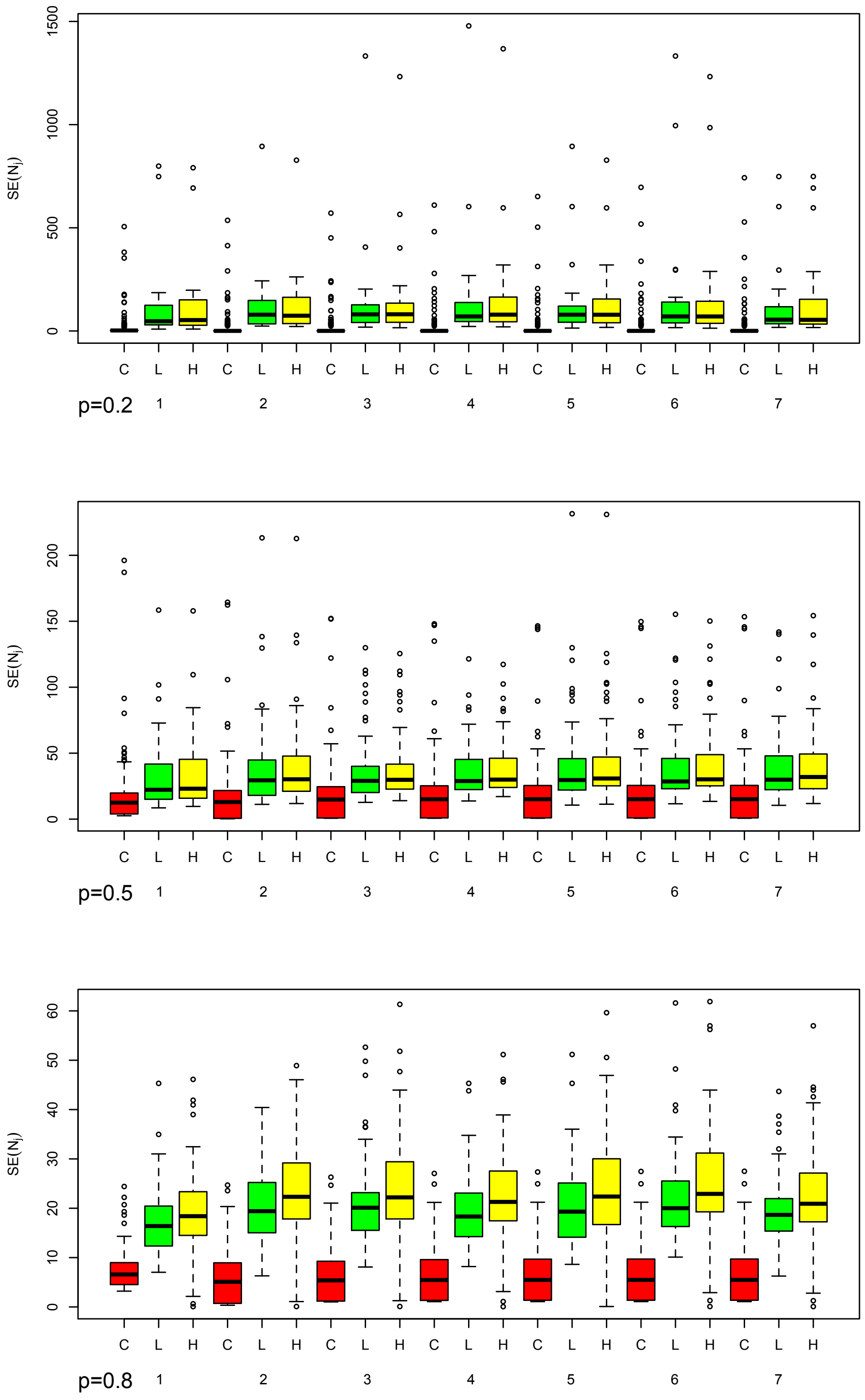

Figure 8: Estimated standard errors for the abundance estimates ($SE(\hat{N}_j)$) for each sample time ($k = 7$) of 100 simulated datasets, for the Crosbie-Manley-Arnason-Schwarz (C: red), the likelihood (L: green), and the pseudo-likelihood (H: yellow; Huggins *et al.* 2010) when parameters values are $N = 200$, and $\phi = 0.2$ for $p = 0.2$ (top), $p = 0.5$ (middle), and $p = 0.8$ (bottom). Estimates from simulations that produced a singular Hessian were removed.

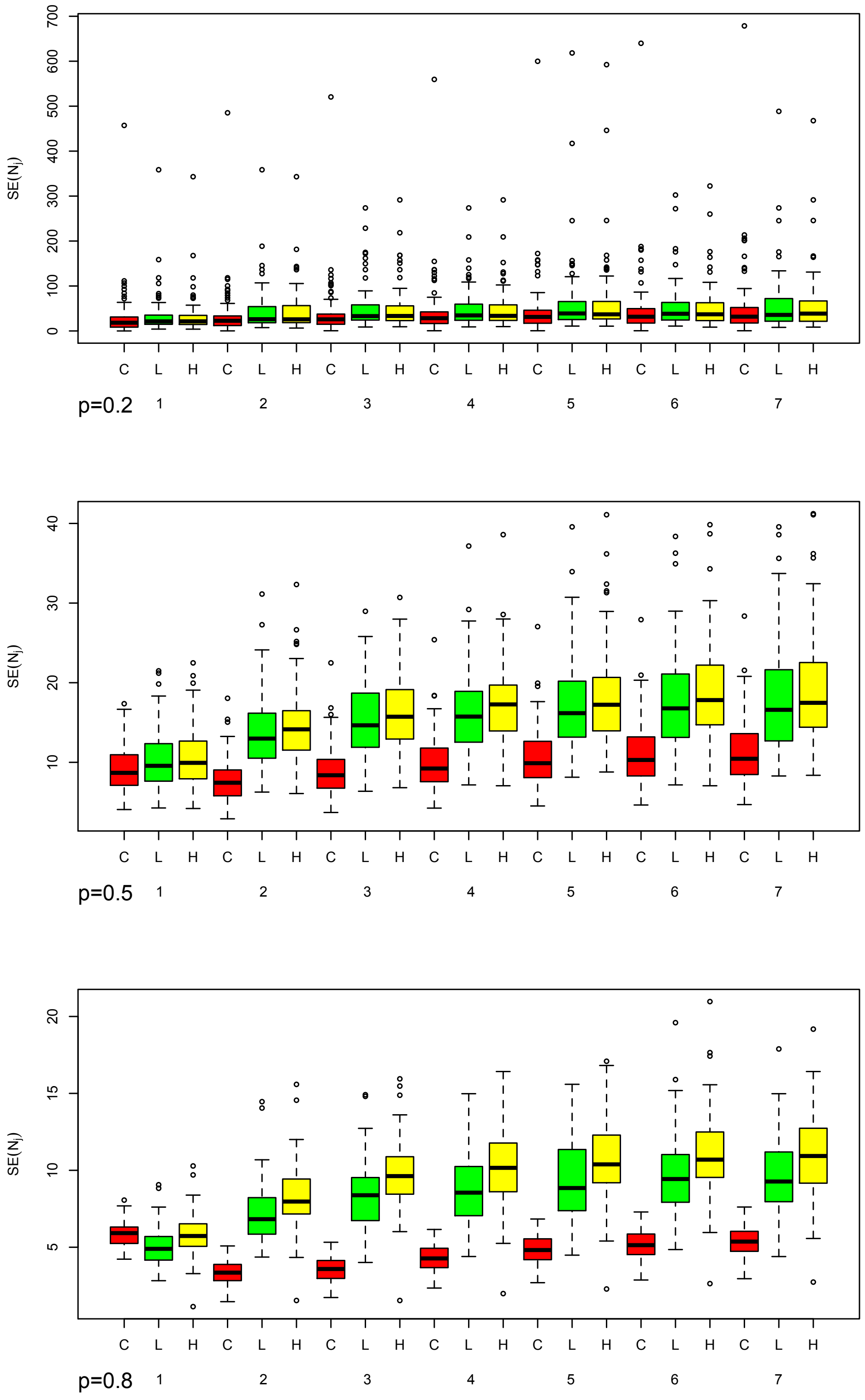

Figure 9: Estimated standard errors for the abundance estimates ($SE(\hat{N}_j)$) for each sample time ($k = 7$) of 100 simulated datasets, for the Crosbie-Manley-Arnason-Schwarz (C: red), the likelihood (L: green), and the pseudo-likelihood (H: yellow; Huggins *et al.* 2010) when parameters values are $N = 200$, and $\phi = 0.5$ for $p = 0.2$ (top), $p = 0.5$ (middle), and $p = 0.8$ (bottom). Estimates from simulations that produced a singular Hessian were removed.

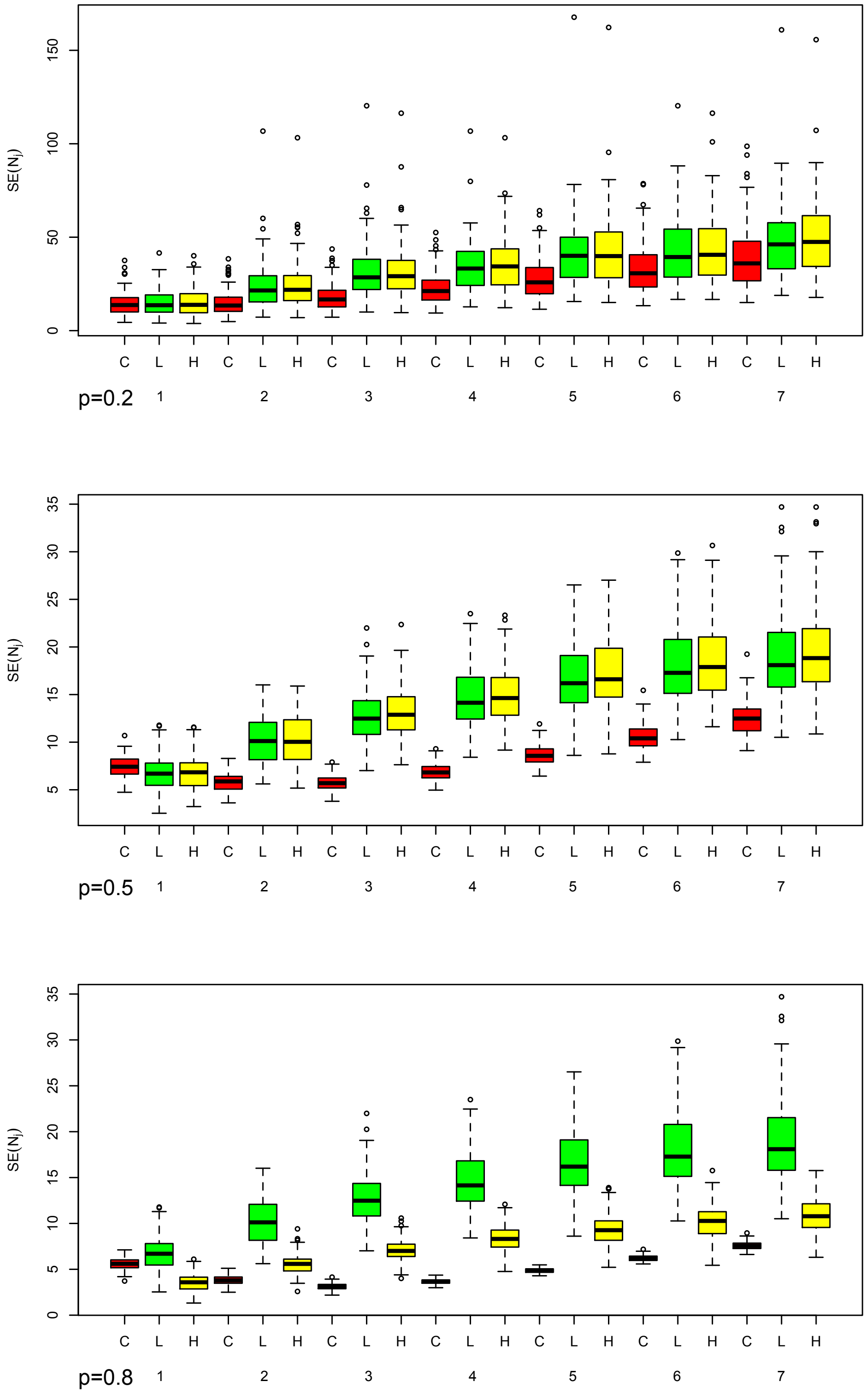

Figure 10: Estimated standard errors for the abundance estimates ($SE(\hat{N}_j)$) for each sample time ($k = 7$) of 100 simulated datasets, for the Crosbie-Manley-Arnason-Schwarz (C: red), the likelihood (L: green), and the pseudo-likelihood (H: yellow; Huggins *et al.* 2010) when parameters values are $N = 200$, and $\phi = 0.8$ for $p = 0.2$ (top), $p = 0.5$ (middle), and $p = 0.8$ (bottom). Estimates from simulations that produced a singular Hessian were removed.

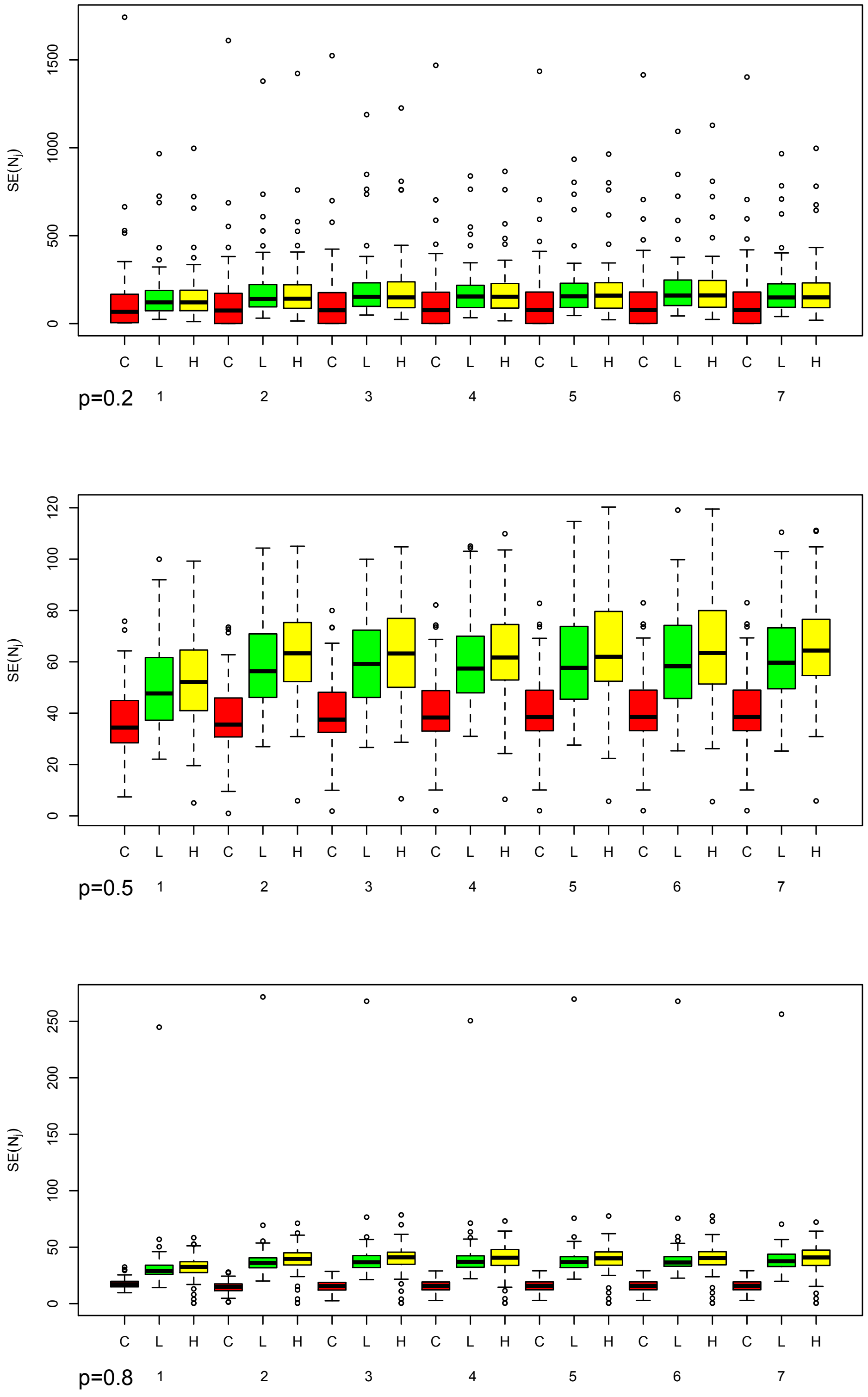

Figure 11: Estimated standard errors for the abundance estimates ($SE(\hat{N}_j)$) for each sample time ($k = 7$) of 100 simulated datasets, for the Crosbie-Manley-Arnason-Schwarz (C: red), the likelihood (L: green), and the pseudo-likelihood (H: yellow; Huggins *et al.* 2010) when parameters values are $N = 1000$, and $\phi = 0.2$ for $p = 0.2$ (top), $p = 0.5$ (middle), and $p = 0.8$ (bottom). Estimates from simulations that produced a singular Hessian were removed.

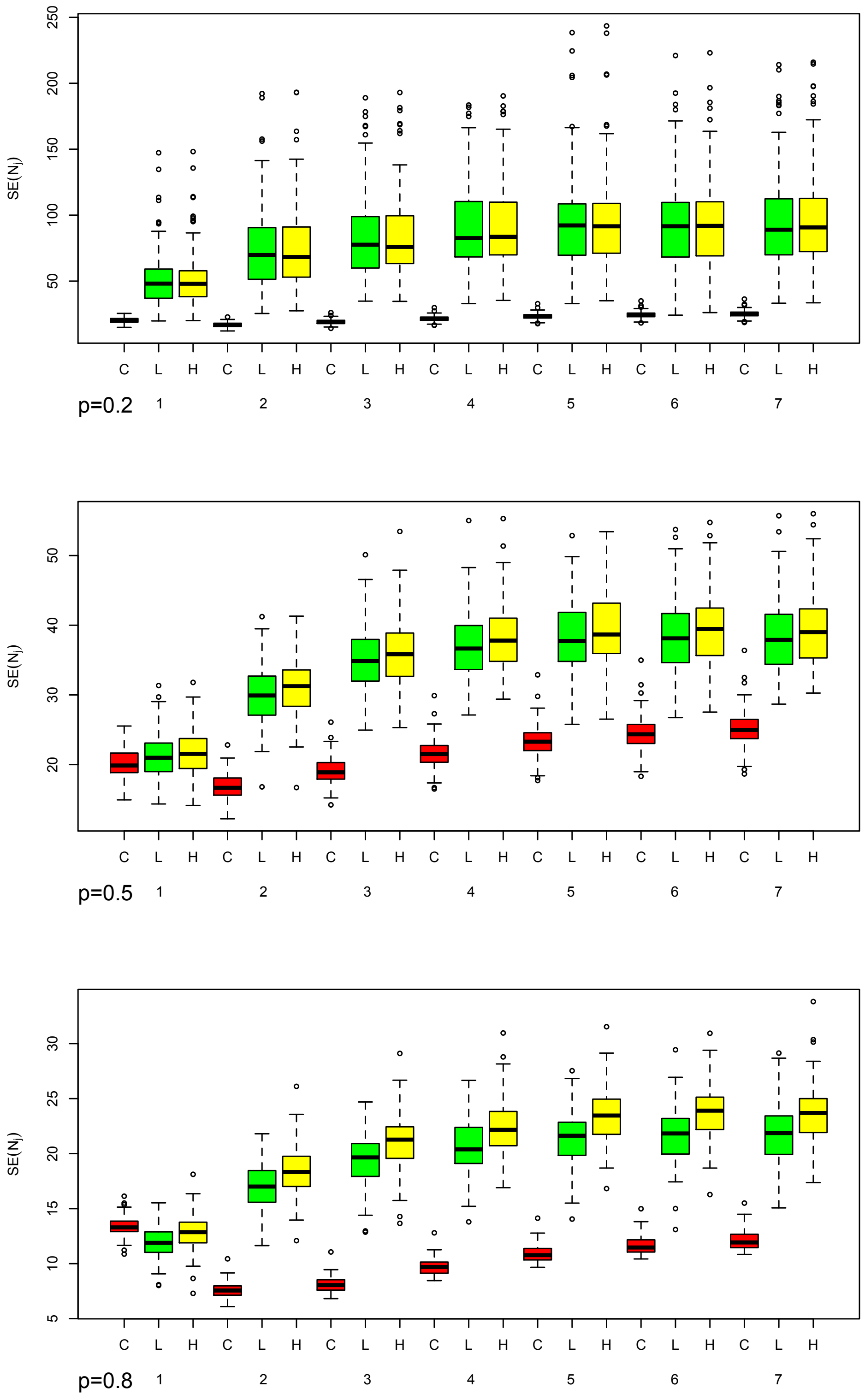

Figure 12: Estimated standard errors for the abundance estimates ($SE(\hat{N}_j)$) for each sample time ($k = 7$) of 100 simulated datasets, for the Crosbie-Manley-Arnason-Schwarz (C: red), the likelihood (L: green), and the pseudo-likelihood (H: yellow; Huggins *et al.* 2010) when parameters values are $N = 1000$, and $\phi = 0.5$ for $p = 0.2$ (top), $p = 0.5$ (middle), and $p = 0.8$ (bottom). Estimates from simulations that produced a singular Hessian were removed.

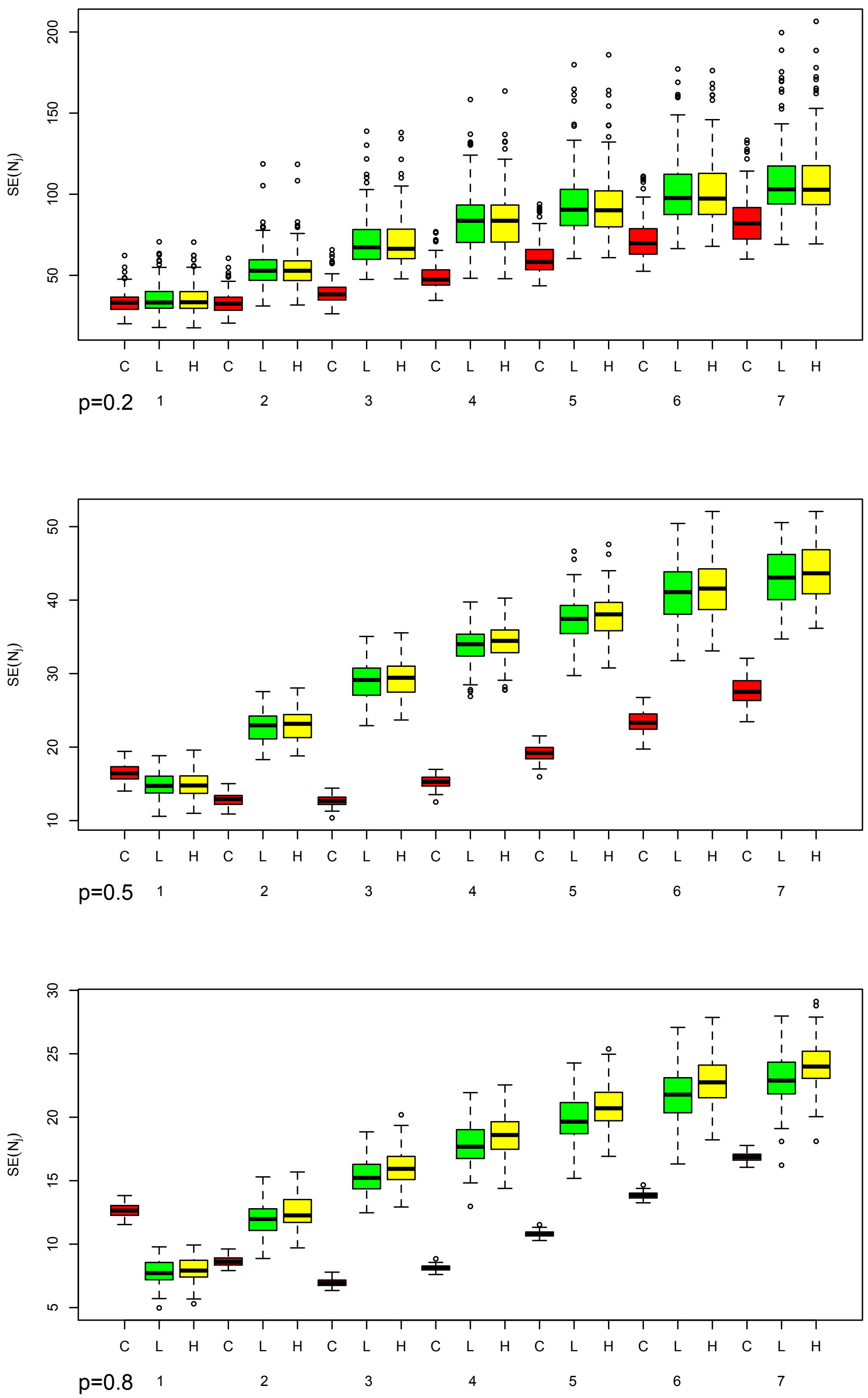

Figure 13: Estimated standard errors for the abundance estimates ($SE(\hat{N}_j)$) for each sample time ($k = 7$) of 100 simulated datasets, for the Crosbie-Manley-Arnason-Schwarz (C: red), the likelihood (L: green), and the pseudo-likelihood (H: yellow; Huggins *et al.* 2010) when parameters values are $N = 1000$, and $\phi = 0.8$ for $p = 0.2$ (top), $p = 0.5$ (middle), and $p = 0.8$ (bottom). Estimates from simulations that produced a singular Hessian were removed.
